# Epidemiological and Genetic Analysis of Foot-and-Mouth Disease Virus O/ME-SA/Ind-2001 in China between 2017 and 2021

**DOI:** 10.1155/2023/3761703

**Published:** 2023-05-19

**Authors:** Xiangle Zhang, Weimin Ma, Fan Yang, Yamin Yang, Lv Lv, Jinyan Wu, Baohong Liu, Chaochao Shen, Yongjie Liu, Zixiang Zhu, Youjun Shang, Jianhong Guo, Xiangtao Liu, Haixue Zheng, Jijun He

**Affiliations:** State Key Laboratory for Animal Disease Control and Prevention, College of Veterinary Medicine, Lanzhou University, WOAH/National Reference Laboratory for Foot-and-Mouth Disease, Lanzhou Veterinary Research Institute, Chinese Academy of Agricultural Sciences, Lanzhou 730000, China

## Abstract

The O/ME-SA/Ind-2001 lineage of foot-and-mouth disease virus (FMDV) was introduced into China in 2017, which subsequently caused 19 outbreaks of foot-and-mouth disease (FMD) and emerged in 8 provinces in China between 2017 and 2021. It is the first time for WOAH/national reference laboratory for FMD (LVRI) to comprehensively summarize these 19 outbreaks of O/ME-SA/Ind-2001 for consecutive 5 years. Our study selected and conducted whole viral genome sequencing for 9 representative isolates and the VP1 sequences of the FMDV-positive samples collected between 2017 and 2021. Analyses of these gene sequences showed that all the field strains belonged to O/ME-SA/Ind-2001e. Phylogenetic analysis indicated that these viruses were closely related to those circulating in neighboring countries, and there were at least 3 different FMD viral clades (named cluster 1, cluster 2, and cluster 3) circulating during this period. Also, a gradually decreasing nucleotide identity was observed in newly emerging viruses year by year compared with the first isolate identified in 2017. These results suggest extensive movements and constant and rapid evolvement of the O/ME-SA/Ind-2001e sublineage. Besides, the neutralizing antigenic sites in the structural proteins for these O/ME-SA/Ind-2001e viruses were analyzed to predict the vaccine matching between these strains and the commercial vaccine strain O/BY/CHA/2010. The results showed that the VP1 epitope 135–144, highly associated with neutralizing antibody induction, was variable among these strains. The mutations in this region reflected a potential risk of challenging the current vaccine protection. Therefore, there is an urgent need to reinforce the importance of improved FMD surveillance and monitor the evolution of O/ME-SA/Ind-2001e, which will provide essential information for the FMD control program in China and Asia.

## 1. Introduction

Foot-and-mouth disease (FMD), caused by foot-and-mouth disease virus (FMDV), is an acute, febrile, and highly contagious disease of cloven-hoofed species, including domestic livestock such as pig, cattle, sheep, and more than 70 wildlife animals [1, 2]. FMD is listed as a “notifiable disease” by the World Organisation for Animal Health (WOAH). The disease is estimated to circulate in 77% of the global livestock population, posing enormous economic losses to the livestock industry and serious socioeconomic damages. The causative agent FMDV belongs to the genus *Aphthovirus* within the family *Picornaviridae*, having seven major viral serotypes (O, A, Asia 1, C, SAT 1, SAT 2, and SAT 3), with no cross-protection between different serotypes as well as insufficient protection between some subtypes within the same serotype. In recent years, new strains of FMDV have continued to arise, which spread rapidly and become threats to the global situation, especially to disease-free areas, resulting in a sustained epidemic and making the etiologic pathogen difficult to control [3, 4].

Serotype O is the most widely prevalent FMDV among the seven serotypes in many parts of the world. More than 80% of outbreaks of FMD in Southeast Asia and East Asia are caused by serotype O. Historically, there are three FMDV lineages of serotype O, which have been circulating in recent years in Southeast Asia and East Asia regions, including O/SEA/Mya-98, O/CATHAY, and O/ME-SA/PanAsia. Another lineage O/ME-SA/Ind-2001, which was first reported and restricted in the Indian subcontinent, has caused large-scale outbreaks of FMD, further complicating the epidemiological situation [5]. O/ME-SA/Ind-2001 is classified into five established, distinct sublineages: a, b, c, d, and e, which account for the majority of outbreaks of current serotype O FMD in countries of the Indian subcontinent [6, 7]. This lineage was detected outside the Indian subcontinent and has spread across the Middle East and North Africa since 2013. In 2015, O/ME-SA/Ind-2001 emerged in Southeast Asia countries, including Lao PDR, Vietnam, and Myanmar [8]. Since then, the O/ME-SA/Ind-2001 lineage gradually spread to Northeast Asia, East Asia, and other neighboring countries. In 2019, O/ME-SA/Ind-2001e was reported in Malaysia, Pakistan, and Cambodia [9, 10]. In May 2022, O/ME-SA/Ind-2001 FMDV emerged in Indonesia, which had been recognized as an FMD-free country since 1990 [11]. Since 2020, there has been an increasing dominance of O/ME-SA/Ind-2001e in Southeast Asia countries, including Cambodia, Laos, Myanmar, Vietnam, and Thailand, as shown at the 24th SEACFMD National Coordinators Meeting (https://rr-asia.woah.org/en/events/24th-seacfmd-national-coordinators-meeting/).

China is thought to have the largest livestock population in Asia. However, the presence of FMD greatly hinders economic development in different regions due to the large population of animals and frequent animal transport. The national reference laboratory for FMD in China first identified the appearance of O/ME-SA/Ind-2001 in 2017. The sequencing and phylogenetic analysis of the VP1 sequence (O/XJ/CHA/2017) revealed that this newly determined strain belongs to O/ME-SA/Ind-2001d and is closely related to strains that have caused FMD outbreaks in Nepal, Myanmar, Russia, and South Korea [12]. Since then, this sublineage has caused increasing outbreaks of FMD. After 2017, sublineage d strains' homology became far from the original sublineage d strains. Hence, the new branch has formed and was later redefined as sublineage e. O/XJ/CHA/2017 was further divided into sublineage e. The increasing dominance of O/ME-SA/Ind-2001e in Pool 1 indicates that FMD epidemiology is characterized by waves of infection sustained by newly emerging O/ME-SA/Ind-2001 lineages supplanting the previous lineages. The objective of this study was to investigate the genetic relationships among different isolates and analyze the genetic diversity of O/ME-SA/Ind-2001e in China and other neighboring countries, which will provide essential information for rearranging the FMD control program in China.

## 2. Materials and Methods

### 2.1. Sample Collection and Outbreaks Investigation

Samples of vesicular fluid, blisters, and tongue or foot epithelium tissue, or OP-fluid, were collected from the livestock in FMDV-affected farms in China. After collection, the samples were immediately transported to the China National Reference Laboratory for FMD (Lanzhou Veterinary Research Institute, LVRI) in an icebox and stored at −80°C for further investigation. This study only focused on the laboratory-confirmed outbreaks caused by O/ME-SA/Ind-2001 during 2017–2021 in China. Sequences of VP1 coding regions of O/ME-SA/Ind-2001 isolates from neighboring countries (Bangladesh, India, Iran, Lao PDR, Mongolia, Myanmar, Nepal, Pakistan, South Korea, Sri Lanka, Thailand, Vietnam, Indonesia, Cambodia, Russia, Bahrain, United Arab Emirates, Kuwait, and Oman) were obtained from GenBank (https://www.ncbi.nlm.nih.gov).

### 2.2. Virus Isolation, RNA Extraction, and cDNA Synthesis

The samples were first detected by real-time RT-qPCR and/or antigen ELISA. Then, the positive samples were cultured on a monolayer of bovine hamster kidney (BHK-21) cells with three successive passages described by the WOAH to obtain the virus isolates. Total RNAs of the collected samples or obtained isolates were extracted using magnetic bead-based RNA extraction kits following the manufacturer's instructions. The extracted RNAs were reverse transcribed into complementary DNA (cDNA) using M-MLV reverse transcriptase (Promega) with random primers according to the manufacturer's protocol.

### 2.3. Whole Genome Sequencing and VP1 Sequencing

As described previously, the representative isolates from several outbreaks were obtained and amplified by PCR using 11 pairs of primers covering the entire FMDV genome [12]. The amplified fragments were sequenced, and SeqMan of the Lasergene package was employed to process and assemble the raw sequence data. FMDV O/XJ/CHA/2017 (GenBank accession No. MF461724) was used as a reference strain. The sequence of the VP1 coding region (639 nucleotides) was amplified using a conventional specific primer pair (1D F: GCGCTGGCAAAGACTTTGA; 1D R: GACATGTCCTCCTGCATCTGGTTGA) as previously described [12], and the fragments were sequenced by Sanger sequencing.

### 2.4. Phylogenetic Analysis

The phylogenetic tree was constructed using ClustalX2 and MEGA X. The maximum likelihood statistical method was employed to conduct the evolutionary history of sequenced isolates in this study and other isolates obtained from GenBank. The general time reversible model (GTR) and gamma distributed with an invariant (*G* + *I*) were used to model evolutionary rate differences among sites. The robustness of tree topology was assessed using 1,000 bootstrap replicates. Initial trees for the heuristic search were obtained by applying the neighbor-joining method to a matrix of pairwise distances estimated using the maximum composite likelihood (MCL) approach. Codon positions included were 1st + 2nd + 3rd + noncoding. All positions containing gaps and missing data were eliminated (complete deletion option).

## 3. Results

### 3.1. Outbreaks of O/ME-SA/Ind-2001 FMD in China since 2017

O/ME-SA/Ind-2001 lineage has been one of the dominant epidemic FMDV lineages since its emergence in 2017. The first confirmed outbreak was identified in January 2017 on a farm in the Hetian region in Xinjiang, northwest China. Four cattle showed clinical signs of FMD. The clinical vesicle fluid or vesicular lesion tissue samples were sent to LVRI in China for viral detection. The samples were detected by real-time RT-PCR, and the results showed that the nucleic acid of the collected samples was FMDV positive. However, the virus was not successfully isolated because of the samples' low amount of live viruses. Then, in February 2017, another outbreak of FMDV infection occurred in the Kashgar region in Xinjiang, with twenty-one cattle showing clinical signs of FMD. All the samples were identified as FMDV serotype O with the same VP1 sequences, and the causative FMDV strain O/XJ/CHA/2017 was isolated and determined as the FMDV O/ME-SA/Ind-2001 lineage virus.

**Figure 1:**
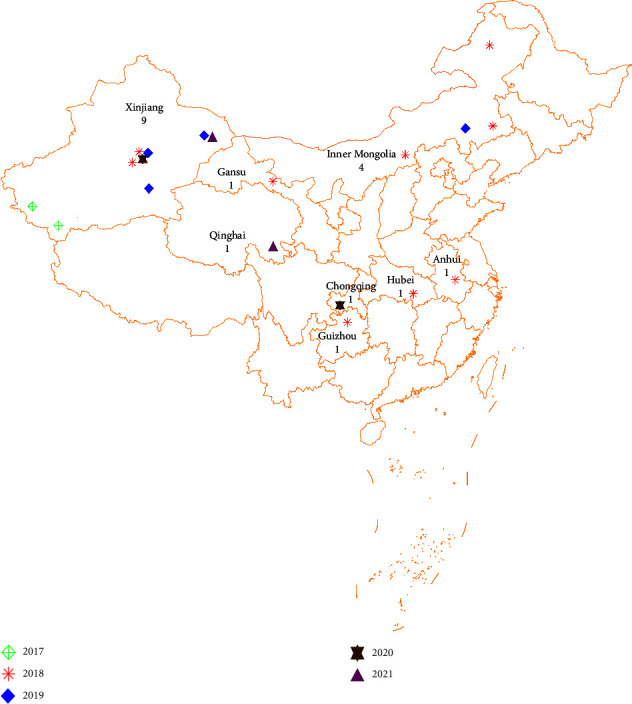
Map of China with the locations where O/ME-SA/Ind-2001 FMD outbreaks were notified from 2017 to 2021 plotted as different icons.

After the first detection of the FMDV O/ME-SA/Ind-2001 lineage in Xinjiang in 2017, this lineage has spread to 8 different provinces or districts between 2017 and 2021 and has caused 19 outbreaks, including 2 outbreaks in 2017, 9 outbreaks in 2018, 4 outbreaks in 2019, 2 outbreaks in 2020, and 2 outbreaks in 2021, respectively. Outbreaks of O/ME-SA/Ind-2001 were reported in China every year within the 5-year study period (Table 1) and were distributed mainly throughout the central and northwestern regions of China (Figure 1), including Xinjiang, Hubei, Anhui, Guizhou, Inner Mongolia, Gansu, Chongqing, and Qinghai. 1648 susceptible animals were affected, and all the cases were reported in cattle. All the infected animals and contact animals were slaughtered after the outbreaks. Epidemiological surveys and studies have shown that the outbreaks in China persistently occurred, mainly caused by long-distance livestock movement (including illegal animal movement).

### 3.2. Genetic Characterization of O/ME-SA/Ind-2001 FMDV Isolates in China

The complete genomes of 9 representative O/ME-SA/Ind-2001 FMDV strains from 9 outbreaks were sequenced. The viral genome ranged from 8202 to 8211 nucleotides (nts) in length with a single large ORF of 6999 nts, which encodes a polyprotein of 2333 amino acids. The complete genome nucleotide identities among these isolates varied from 94.8% to 99.4%, as reflected in the pairwise sequence comparison. A gradually decreasing nucleotide identity was observed in newly emerging viruses year by year compared with the isolate identified in 2017 (O/XJ/CHA/2017). The strains isolated between 2020 and 2021 had the lowest nucleotide identity with O/XJ/CHA/2017.  Table 2 lists the identity of the nucleotide and related amino acid of the single large ORF, each structure protein, nonstructure protein, UTRs, and the whole genome in the 9 sequencing sequences compared with the O/XJ/CHA/2017 strains.

**Figure 2:**
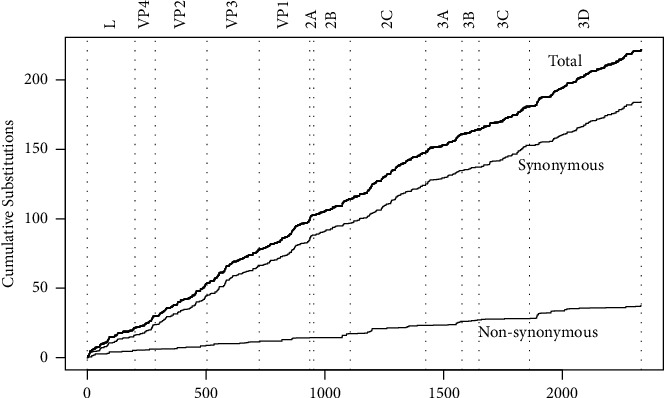
Graphic representation of cumulative total, synonymous, and nonsynonymous substitutions against per site across the ORF of O/ME-SA/Ind-2001 FMDV in China as estimated using SNAP. The *X*-axis represents the amino acid position of the ORF, and the *Y*-axis represents cumulative total/nonsynonymous/synonymous substitutions against amino acid positions as estimated using SNAP.

The phylogenetic analysis showed that the ORF homology of sequenced virus strains shared 95.9–97.6% nucleotide identity and 97.5–98.8% amino acid identity with O/XJ/CHA/2017, respectively. The further analysis detected 870 substitution sites throughout the ORF region among the aligned sequences, of which 309 were nonsynonymous substitutions. 103 amino acid differences were distributed into 11 proteins, including L (12), VP4 (3), VP2 (7), VP3 (10), VP1 (9), 2B (3), 2C (16), 3A (10), 3B (4), 3C (3), and 3D (26) (Table 2). The amino acid sequence encoding for 2A was found to be conserved. No nucleotide insertions or deletions were present between these genomes ORF. The nonsynonymous substitutions per site were estimated using SNAP (https://www.hiv.lanl.gov/content/sequence/SNAP/SNAP.html) (Figure 2).

### 3.3. Phylogenetic Analysis

The VP1 sequences of the O/ME-SA/Ind-2001 strains isolated in China and 91 O/ME-SA/Ind-2001 strains isolated in other 20 countries (7 from Indonesia, 3 from Cambodia, 7 from Vietnam, 2 from Thailand, 1 from Mongolia, 4 from South Korea, 6 from Myanmar, 2 from Russia, 10 from Nepal, 5 from Bangladesh, 3 from Bahrain, 8 from Bhutan, 1 from Lao PDR, 24 from India, 3 from Pakistan, 1 from Iran, 1 from Sri Lanka, 1 from the United Arab Emirates, 1 from Kuwait, and 1 from Oman) were aligned. The results showed that all the collected strains in China belonged to the O/ME-SA/Ind-2001e sublineage. The strains from the 19 outbreaks in China formed into three genetic clusters within the O/ME-SA/Ind-2001e sublineage. The homology of the two strains isolated in 2017 was 99.1%. The strains isolated in 2018 shared 96.1–98.4% identity with that in 2017. The strains isolated in 2019 were 96.2–97.2% homology to that in 2017. The strains isolated in 2020 were 95.9–96.7%, and those isolated in 2021 were 95.1–96.1% compared to 2017. These results indicated that the strains isolated in 2020 and 2021 differed notably from previous O/ME-SA/Ind-2001 strains in China.

**Figure 3:**
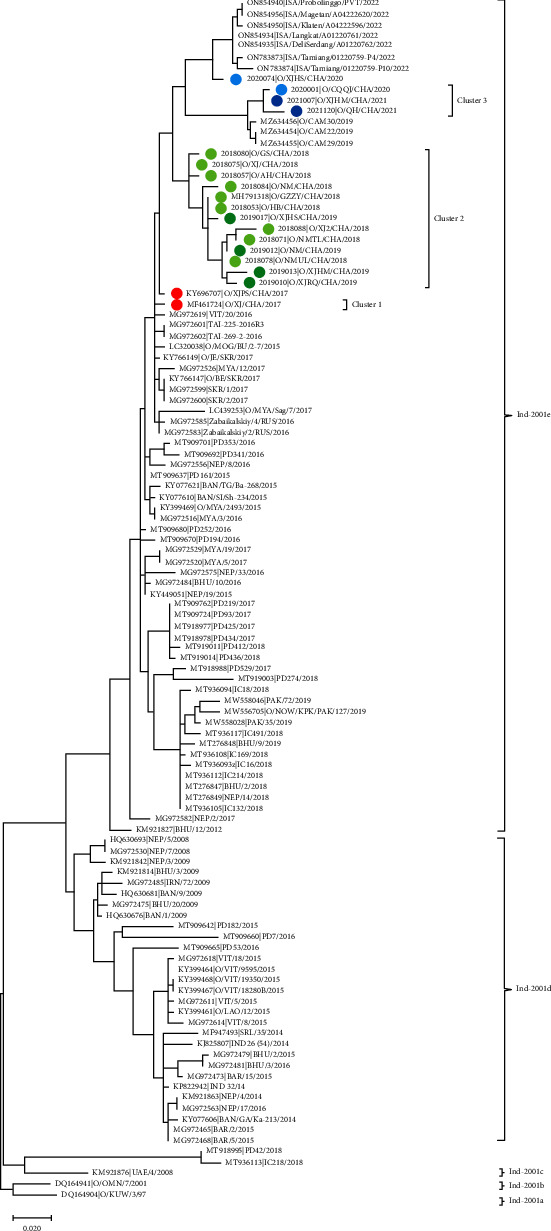
The maximum likelihood tree based on viral VP1 sequences shows the relationships between FMDV O/ME-SA/Ind-2001e isolates collected from China in 2017–2021 and other countries. The sequence generated in this study is shown with different colors of the circle symbol.

The O/ME-SA/Ind-2001 sublineage strains isolated in 2017 in China were classified into cluster 1 in this study. These strains were closely related to the strains from South Korea, Myanmar, Thailand, and Mongolia that were determined before 2017 (having homology from 97.7% to 99.5%), suggesting that the O/ME-SA/Ind-2001 sublineage was introduced into China from neighboring countries. However, we still need more epidemiological information to identify the initial source of the outbreak. The strains isolated in 2018 and 2019 were listed as another cluster defined as cluster 2. The strains isolated between 2020 and 2021 were homologous to Cambodia and Indonesia and classified as cluster 3.  Figure 3 shows the phylogenetic tree of FMDV O/ME-SA/Ind-2001e isolates collected from China and other countries. The strains in cluster 2 were closely related to viruses previously circulating in 2017 (cluster 1). However, these strains were subsequently replaced by the strains in cluster 3 in 2020 and 2021.

### 3.4. Antigen Variability Analysis of O/ME-SA/Ind-2001e Strains in China

**Figure 4:**
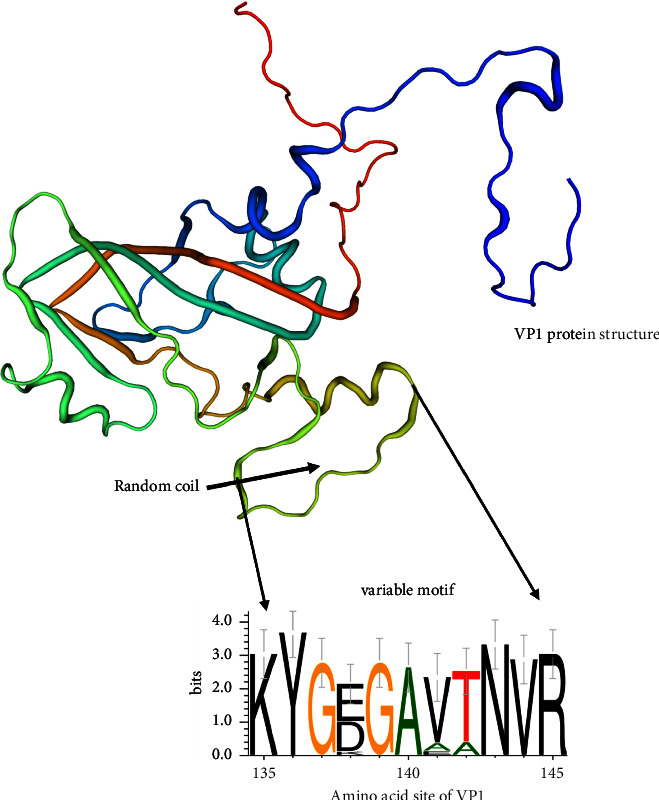
The structure of FMDV VP1 protein and the variable motif (135–144 region) of sequence alignment in 19 VP1 protein. The data were shown as WebLogo, which generates sequence logos and reveals significant alignment features (https://weblogo.berkeley.edu/logo.cgi) [14].

FMDV has a high mutation frequency in natural evolution. For serotype O FMDV, the critical residues in five neutralizing antigenic sites (B cell epitopes) were identified in VP1 (43–51, 135–144, 198, and 206–208), VP2 (70–77 and 131–134), and VP3 (56–79) proteins, which may be related to vaccine selection and virus evolution [4, 13]. To compare the antigenic sites between O/ME-SA/Ind-2001e strains and the current commercial vaccine strain O/BY/CHA/2010 (JN998085), the variability of the antigenic sites in VP2 and VP3 proteins in 10 sequenced strains and VP1 protein in 19 strains was evaluated and compared with the vaccine strain. We found that there are 11 important amino acid changes: 5 in VP1 protein (138 G/E/D/K, 140 S/A, 141 L/V/E, 142 P/T/A, and 198A/Q), 4 in VP2 protein (79 Y/H, 130 C/Y, 133 E/Q, and 134 R/K), and 2 in VP3 protein (58 D/E and 67 K/R). Most of the amino acid changes were sporadic, whereas the 135–144 region in VP1 was the most variable among these strains, as shown in Figure 4. The mutation of the 135–144 region might not affect the whole VP1 structure, but this region is highly associated with neutralizing antibody induction, reflecting a potential risk of evading current vaccine coverage.

## 4. Discussion

FMD has been reported in China since 1958 [15]. The emergence of novel subtypes complicates the epidemiology and control measures of FMDV. China reported 175 outbreaks of FMD to the WOAH between 2005 and 2021. The detection results of FMDV samples in China during 2005–2021 showed that serotype Asia 1 had not been detected since 2009, that serotype A tends to be under control through effective immunization programs, and that serotype O is currently the most prevalent FMDV serotype with different topotypes or lineages (such as O/Mya-98, O/PanAsia, O/Ind-2001, and O/CATHAY) circulating simultaneously, suggesting that the epidemic situation of FMD in China will continue to be dominated by sporadic diversified FMDV serotype O.

The risk of exogenous strains' introduction into China still exists, which may affect the current epidemic. In China, the outbreak of FMD caused by the O/ME-SA/Ind-2001 lineage was first reported in 2017, and then, new outbreaks occurred every year for five consecutive years. The traceability analysis performed by LVRI and the World Reference Laboratory for Foot-and-Mouth Disease (WRLFMD) showed that the homology of the virus strain O/XJ/CHA/2017 was 99.2–99.7% with that in Nepal (NEP/19/2015 and NEP/6/2016 strains), Vietnam (2016), and South Korea (2017), suggesting that these FMDV O/ME-SA/Ind-2001 strains in China and Southeast Asia shared a high nucleotide identity. These strains might originate from one ancestor. According to the epidemic dynamics of this lineage abroad, combined with the historical situation in China, we speculated that this strain previously spread around Southeast Asia and then introduced into China, or it spread around the northern countries of China and then introduced into the region along the northwest or northeast direction in China.

Furthermore, based on the SEACFMD database, nearly one million illegally traded cattle from Southeast Asian countries entered the Chinese market every year, which provided an opportunity to spread FMDV and added the possibility of introducing this strain into China from Southeast Asia. Traceability analysis of O/GZZY/CHA/2018 (MH791318) in cattle showed that the homology of this strain was 97.3–97.7% with that in Myanmar (2017), Bangladesh (2015), and South Korea (2017). The strains collected in 2020 and 2021 had 95.4–95.7% homology with O/GZZY/CHA/2018, which implied that O/ME-SA/Ind-2001e FMDV tends to evolve into new branches after its introduction.

Vaccination is an important strategy to prevent FMDV spread [16]. Several vaccines, including O-Manisa, O-3039, and O-TUR/5/2009, are widely used in FMD vaccine-antigen reserves and might provide high protection against O/ME-SA/Ind-2001 lineages. However, cattle vaccinated with these vaccines were not fully protected against the O/ME-SA/Ind-2001 lineage viruses in North Africa and South Korea [17, 18], indicating their unsuitability to provide sufficient protection against O/ME-SA/Ind-2001 viruses. The possible reason is the low-matching of the antigenic relationship (*r*1 value) depending on isolates. When the first strain was isolated in China, a virus neutralization test (VNT) was carried out for the type O vaccine against O/XJ/CHA/2017. The results showed that the vaccine has a high neutralization titer against this strain after primary vaccination and boost vaccination, inferred to be immune protective (data not shown). However, our study found that the O/ME-SA/Ind-2001e strains in 2017 have been replaced by the strains distributed in a new cluster (cluster 3 in this study). There is a tendency for O/ME-SA/Ind-2001 virus strains to evolve into new branches. Genetic variability and antigenic diversity in capsid coding regions, especially VP1 protein, might result in the FMDV' antigenic variation, evading the host immunity [19, 20]. Besides, whether the persistent epidemic of O/Ind-2001 lineage is related to quasi-species emergence under the pressure of vaccine immunization, susceptible animal species, or the pathogen ecology of FMDV remains unknown. Therefore, it is important to constantly monitor capsid coding regions of field strains and measure the efficacy of the current vaccine strains against new O/ME-SA/Ind-2001e isolates in cattle or other livestock.

In conclusion, the findings of this study demonstrate that the circulating FMDV O/ME-SA/Ind-2001 strains from 2017 to 2021 in China all belong to sublineage e, and this lineage was introduced to China from aboard, revealing the characteristics of regional transmission of emerging and transboundary animal diseases. The isolated strains in 2020 and 2021 showed a significant genetic variation from the previously reported O/ME-SA/Ind-2001e strains in China. This sublineage's constant and rapid evolvement may result in the evasion of the current commercial vaccine coverage and an increase in FMD outbreaks. The findings of our study highly recommend the ongoing studies and surveillance of the evolution of O/ME-SA/Ind-2001e. In the future, international exchanges and cooperation are needed and should be further strengthened in research and prevention and control of transboundary animal diseases or new emerging animal diseases, which might promote regional prevention, control, purification, and eradication of important animal diseases.

## Figures and Tables

**Table 1:** Overview of the FMDV O/ME-SA/Ind-2001 outbreaks in China since 2017.

Year	Report date	Species	Susceptible	Cases	Deaths	Destroy	Location	Province	Isolate	Length
2017	11/01/2017	Cattle	6	4	0	6	Hetian	Xinjiang	**KY696707** [12]	—
2017	14/02/2017	Cattle	42	21	0	42	Marabishi	Xinjiang	**MF461724** [12]	8211
2018	19/05/2018	Cattle	79	11	2	77	Jingzhou	Hubei	2018053	8210
2018	12/06/2018	Cattle	63	16	11	52	Wuhu	Anhui	2018057	—
2018	25/06/2018	Cattle	82	15	0	82	Zunyi	Guizhou	MH791318	—
2018	30/07/2018	Cattle	149	11	0	149	Tongliao	Inner Mongolia	2018071	—
2018	23/08/2018	Cattle	175	63	4	171	Weili	Xinjiang	2018075	8204
2018	29/08/2018	Cattle	112	36	12	100	Ulanqab	Inner Mongolia	2018078	—
2018	14/09/2018	Cattle	47	8	0	47	Zhangye	Gansu	2018080	—
2018	19/10/2018	Cattle	140	17	0	140	Hulunbuir	Inner Mongolia	2018084	8210
2018	20/11/2018	Cattle	74	13	0	74	Heshuo	Xinjiang	2018088	8206
2019	22/02/2019	Cattle	85	45	35	50	Balinzuoqi, Chifeng	Inner Mongolia	2019012	8202
2019	16/04/2019	Cattle	66	11	0	66	Heshuo	Xinjiang	2019017	8208
2019	06/06/2019	Cattle	286	9	0	286	Ruoqiang	Xinjiang	2019010	—
2019	13/08/2019	Cattle	67	2	0	67	Hami	Xinjiang	2019013	—
2020	01/06/2020	Cattle	12	4	0	12	Qijiang district	Chongqing	2020001	8206
2020	17/10/2020	Cattle	70	6	0	70	Heshuo	Xinjiang	2020074	—
2021	29/01/2021	Cattle	52	37	0	52	Hami	Xinjiang	2021007	8205
2021	31/10/2021	Cattle	41	40	0	41	Zeku	Qinghai	2021120	8210

**Table 2:** The homology comparisons of nucleotides and amino acids between O/ME-SA/Ind-2001 FMDV strains and the MF461724 strain.

Strains	5′UTR	L (12)	P1				P2			P3							ORF (103)	3′UTR	Genome
			VP4 (3)	VP2 (7)	VP3 (10)	VP1 (9)	2A (0)	2B (3)	2C (16)	3A (10)		3B (4)		3C (3)	3D (26)				
2018053	96.9	96.7	97.3	97.2	97.3	97.0	93.8	97.6	97.2	98.0		99.5		97.7	98.0		97.5	97.3	97.5
		(98.0)	(98.8)	(98.6)	(99.5)	(98.1)	(100.0)	(98.7)	(99.4)	(98.0)		(100.0)		(100.0)	(98.3)		(98.8)		

2018075	97.2	97.5	96.9	97.1	96.8	98.1	93.8	97.4	97.3	98.5		99.5		97.2	97.9		97.6	96.2	97.6
		(97.5)	(98.8)	(98.6)	(99.5)	(99.1)	(100.0)	(98.7)	(98.4)	(98.7)		(100.0)		(99.5)	(98.3)		(98.8)		

2018084	96.0	96.5	97.6	96.5	96.8	97.2	93.8	97.4	96.5	97.6		99.1		96.7	97.9		97.2	97.3	97.1
		(98.0)	(98.8)	(98.6)	(99.5)	(99.1)	(100.0)	(98.7)	(99.1)	(98.0)		(100.0)		(100.0)	(97.7)		(98.8)		

2018088	96.3	96.2	96.9	96.6	96.8	96.1	93.8	97.4	97.0	97.8		99.1		97.2	97.5		97.0	97.2	97.0
		(97.5)	(98.8)	(99.1)	(99.5)	(98.1)	(100.0)	(98.7)	(99.1)	(98.7)		(100.0)		(100.0)	(98.1)		(98.8)		

2019012	95.7	95.9	96.9	96.5	95.6	96.7	91.7	97.4	96.8	98.0		98.6		97.5	97.4		96.9	97.1	96.9
		(97.5)	(98.8)	(98.6)	(99.5)	(98.6)	(100.0)	(98.7)	(98.7)	(98.7)		(100.0)		(100.0)	(97.7)		(98.7)		

2019017	96.0	96.0	96.5	96.5	96.4	96.9	93.8	97.2	96.5	97.6		99.5		97.2	97.4		97.0	96.4	97.0
		(97.5)	(96.5)	(99.1)	(99.1)	(98.6)	(100.0)	(98.7)	(98.4)	(98.7)		(100.0)		(100.0)	(97.2)		(98.5)		

2020001	93.7	96.5	95.7	95.7	95.6	95.9	91.7	97.0	95.7	95.9		96.2		97.5	95.7		96.2	97.2	96.0
		(97.5)	(100.0)	(99.1)	(98.2)	(99.1)	(100.0)	(98.1)	(97.5)	(97.4)		(95.8)		(99.1)	(98.1)		(98.2)		

2021 007	93.9	96.4	95.7	95.9	95.9	96.1	91.7	97.8	95.3	96.1	96.7		97.7			95.7	96.2	97.2	96.0
		(97.5)	(100.0)	(99.1)	(98.6)	(99.1)	(100.0)	(98.1)	(96.9)	(97.4)	(95.8)		(99.1)			(97.7)	(98.1)		

2021120	93.7	95.2	96.1	95.4	95.0	95.1	93.8	97.4	95.1	95.9	96.2		97.8			95.7	95.9	97.3	95.7
		(95.5)	(100.0)	(98.2)	(96.4)	(97.7)	(100.0)	(98.1)	(96.9)	(97.4)	(94.4)		(99.1)			(97.7)	(97.5)		

*Note.* Nucleotide identity (%) (amino identity (%)); () represents the number of total nonsynonymous substitutions distributed in each gene.

## Data Availability

The data supporting this study's findings are available from the corresponding authors upon reasonable request.
